# Supplementary material for: Revealing the Critical Role of Global Electron Density Transfer in the Reaction Rate of Polar Organic Reactions within Molecular Electron Density Theory
Source: Molecules. 2024 Apr 19;29(8):1870. doi: 10.3390/molecules29081870 (PMC11053847; doi:10.3390/molecules29081870)
Supplement: Supplementary file 1 [file molecules-29-01870-s001.zip › molecules-2961399-supplementary.pdf]

Supplementary Material

Revealing the Critical Role of Global Electron Density Transfer in the Reaction Rate of Polar Organic Reactions within Molecular Electron Density Theory

Luis R. Domingo * and Mar Ríos-Gutiérrez *

Department of Organic Chemistry, University of Valencia, Dr. Moliner 50, 46100 Burjassot,Valencia, Spain
* Correspondence: luisrdomingo@gmail.com (L.R.D.); m.mar.rios@uv.es (M.R.-G.)

Index

S3 The Interacting Quantum Atom scheme
S4 ELF and QTAIM topological analysis of the TSs.
S10 References.
S12 **Figure S4** with the plot of the computed M06-2X/6-311G(d,p) activation Gibbs free energies versus the number of cyano groups on the ethylene for the DA reactions of Cp **1** with the cyanoethylene series **2**.
S13 **Figure S5** with the plot of the logarithm of the experimental relative reaction rate constants versus the logarithm of the computed M06-2X/6-311G(d,p) relative reaction rate constants for the DA reactions of Cp **1** with the cyanoethylene series **2**.
S14 **Figure S6** with the M06-2X/6-311G(d,p) *in-vacuo* optimized geometries of the TSs involved in the DA reactions of Cp **1** with ethylene **4** and the cyanoethylene series **2**.
S15 **Figure S7** with the plot of the *in-vacuo* GEDT obtained by using Bader charges with respect to *in-vacuo* GEDT obtained by using NPA charges.
S16 **Figure S8** with the plot of the logarithm of the experimental reaction rate constants versus the IQA stabilization of the ethylene framework for the DA reactions of Cp **1** with the cyanoethylene series **2**.
S17 **Table S2** with the M06-2X/6-311G(d,p) *in-vacuo* total electronic energies of reagents, TSs and CAs involved in the DA reactions of Cp **1** with ethylene **4** and the cyanoethylene series **2**.
S18 **Table S3** with the M06-2X/6-311G(d,p) enthalpies, entropies and Gibbs free energies, computed at 293.15 K and 1 atm in dioxane, for the stationary points involved in the DA reactions of Cp **1** with ethylene **4** and the cyanoethylene series **2**.

S19 **Table S4** with distances between the C1–C6 and C2–C3 interacting carbons at the M06-2X/6-311G(d,p) optimized TSs in dioxane, geometrical asynchronicity, average of the two C–C interacting distances, and GEDT values.

S20 **Table S5** with the total intra- and interatomic IQA energies at the diene and ethylene frameworks of the TSs and at the reagents involved in the DA reactions of Cp **1** with ethylene **4** and the cyanoethylene series **2**.

S21 M06-2X/6-311G(d,p) *in-vacuo* computed total energies, single imaginary frequencies of TSs, and Cartesian coordinates of the stationary points involved in the DA reactions of Cp **1** with ethylene **4** and the cyanoethylene series **2**.

The Interacting Quantum Atom scheme

The Interacting Quantum Atom [1] (IQA) scheme based on quantum theory of atoms in molecules [2,3] (QTAIM) divides the total energy into two main energy contributions: the intra-atomic energy, E_{intra}^{A}, and the interatomic energy, V_{inter}^{AB}. The interatomic energy is, in turn, divided into two additional terms: the interatomic electrostatic energy (typically referred to as "classical"), V_{cl}^{AB}, and the interatomic exchange-correlation energy, V_{xc}^{AB}, in such a way that $V_{inter}^{AB} = V_{cl}^{AB} + V_{xc}^{AB}$. While E_{intra} has been associated with steric effects [4], V_{cl} is related to electrostatic interactions [5,6], and V_{xc} quantifies covalency [7].

A straightforward extension of IQA, called Interacting Quantum Fragments (IQF) [8], is used thanks to the additivity of topological atoms. IQF consists on grouping IQA energy terms of convenient fragments of the system to allow for a more chemically meaningful analysis of the interactions taking place between groups of atoms. In this study, we have chosen the two interacting Cp and cyanoethylene frameworks (X = Cp or Et). By default, we consider the sum of all atomic energies belonging to the considered fragment. Relative energies are analyzed by difference between the total IQA energies of each of the two interacting frameworks (X) at the TSs and the corresponding total IQA energies computed at the GS of the reagents. Thus, for the sake of clarity, the following abbreviations have been used in the manuscript: relative total IQA energies ($\sum \Delta E_{IQA}^{X}$) as E_{tot}(X), relative total intra-atomic ($\sum \Delta E_{intra}^{A}$) as E_{intra}(X), relative total interatomic ($\sum \Delta V_{inter}^{X}$) as V_{inter}(X), the total interatomic interactions between the two Cp and Et fragments ($\sum V_{inter}^{AB}$(Cp,Et)) as V_{inter}(Cp,Et), and the relative total interatomic energies within the two Cp and ethylene frameworks ($\sum \Delta V_{inter}^{AB}$(X)), i.e. without considering V_{inter}(Cp,Et), are labelled V'_{inter}(X). Note that V_{inter}(X) = V'_{inter}(X) + V_{inter}(Cp,Et) and therefore, E'_{tot}(X) = E_{intra}(X) + V'_{inter}(X). The relative total, intra- and interatomic IQA energies are a measure of how much the reagents are destabilized (positive relative energies) or stabilized (negative relative energies) when going from their GS to the TS. Thus, the sum of the relative total IQA energies of the two interacting frameworks, E_{tot}(Cp+Et), i.e. the sum of the energy variation of the two fragments, yields the electronic IQA activation energy of these P-DA reactions.

ELF and QTAIM topological analysis of the TSs

The *in-vacuo* electronic structures of the seven TSs were analyzed by electron localization function [9] (ELF) and QTAIM [2,3] topological analyses. The ELF basin attractors together with the most relevant valence basin populations of the reagents and the TSs are given in Figures S1 and S2, respectively.

Topological analysis of the ELF of the cyanoethylene series shows that the presence of the cyano groups on the six ethylene derivatives does not substantially modify the ELF description of the C1–C2 bonding region (see Figure S1). It is characterized by the presence of two V(C1,C2) disynaptic basins, integrating a total population of ca. 3.30 e, thus being associated with an underpopulated double bond within the Lewis bonding model.

ELF topological analysis of **TS-Et** shows a similar behavior to that of the TS associated with the N-DA reaction between butadiene and ethylene (see Figure S2) [10,11]. In all TSs, while the C1–C2, C3–C4 and C5–C6 bonding regions have been depopulated, being characterized by the presence of only one V(Ci,Cj) disynaptic basin, the C4–C5 has been slightly populated. Note that these C3–C4 and C5–C6 bonding regions at Cp **1** are characterized by the presence of two pairs of V(Ci,Cj) disynaptic basins, integrating a total population of 3.40 e each pair (see Figure S1). These bonding changes, which take place in all DA reactions, are required for the subsequent formation of the *pseudoradical* centers [12], which are demanded for the formation of the new C–C single bonds [13]. No V(Ci) monosynaptic basin associated with the formation of the corresponding *pseudoradical* centers is observed at **TS-Et** (see Figure S2).

Interestingly, the polar TSs **TS-1CN**, **TS-2cCN** and **TS-2tCN** show the same behaviors as the non-polar **TS-Et** (see **TS-Et** and **TS-1CN** in Figure S2). Consequently, the presence of one cyano group or two vicinal cyano group in the symmetrically substituted **2cCN** and **2tCN** does not substantially modify the electronic structures of these TSs with respect to the non-polar **TS-Et**.

A different behavior is found at the more polar **TS-2CN** – **TS-4CN**. The asynchronous **TS-2CN** and **TS-3CN** are characterized by the presence of two monosynaptic basins, V(C1) and V(C6), integrating less than 0.34 e, while the synchronous **TS-4CN** is characterized by the presence of two monosynaptic basins, V(C1) and V(C2), integrating 0.42 e each, at the ethylene framework (see **TS-2CN** and **TS-4CN** in Figure S2). These ELF behaviors found at the more polar **TS-2CN** – **TS-4CN**

are a consequence of the high GEDT taking place at the corresponding TSs [13] which, according the analysis of the electrophilic P_k^+ Parr functions at the corresponding cianoethylenes, will mostly accumulate at the C1 and C2 carbons (see Figure 2).

The seven TSs show the absence of any V(C1,C6) or V(C2,C3) disynaptic basin, indicating that formation of the new C1−C6 and C2−C3 single bonds has not yet begun in any TS, in agreement with the geometrical analysis of the TSs.

The highly asynchronous **TS-2CN** is associated with a non-concerted *two-stage one-step* mechanism [14] in which the formation of the second C2−C3 begins when the first C1−C6 single bond is practically formed. ELF of the TSs given in Figure S2 shows that no TS, including the symmetric **TS-Et** and **TS-4CN**, cannot be associated with a concerted process as proposed for the "pericyclic" mechanism in 1969 by Woodward and Hoffman [15], and also widely supported by Houk [16]. According to Domingo's model for Ci−Cj single bond formation in organic reactions involving C−C multiple bonds [13], the electron density required for the C-to-C coupling of the two *pseudoradical* centers Ci and Cj comes from the previous depopulation of the C−C double bonds of the reagents. These DA reactions begin with the rupture of the three C−C double bonds, and end with the formation of the C−C double bond present in the final cyclohexene [11]. Consequently, as formation of the new C−C single bond requires the prior rupture of the C−C double bonds, the bonding changes along a cycloaddition cannot take place simultaneously (i.e. concerted), but sequentially. Reactions that take place through cyclic TSs, where it is not necessary that all neighboring atoms are bound, have been classified as *pseudocyclic* reactions [17].

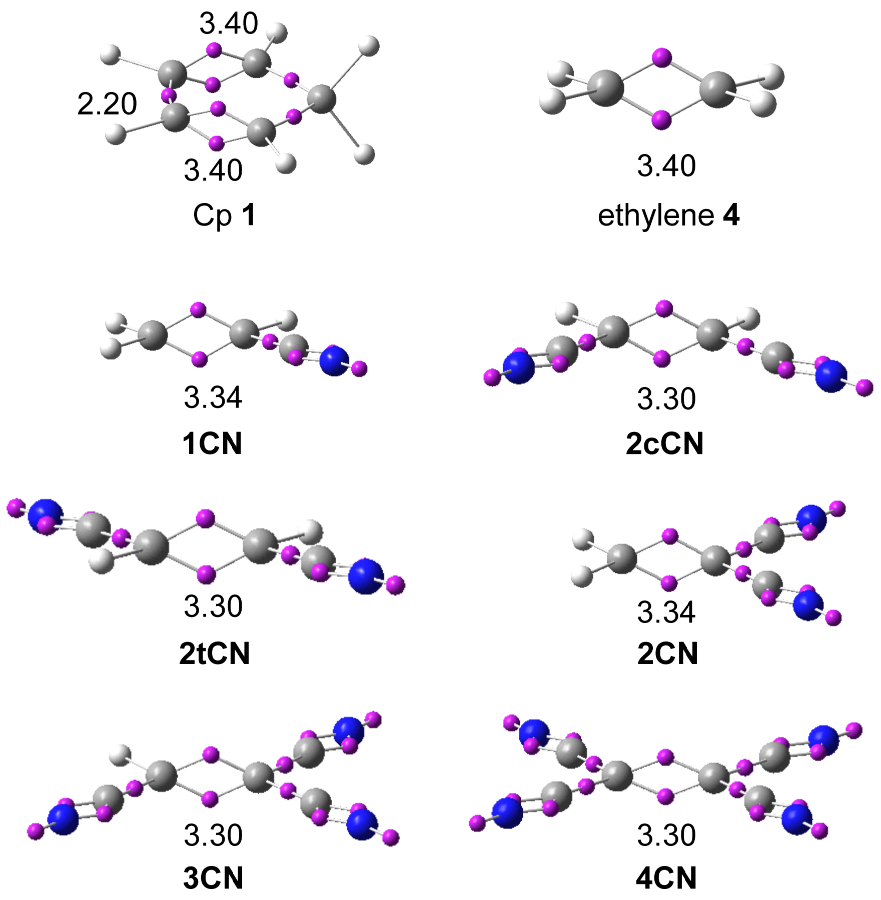

Figure S1. M06-2X/6-311G(d,p) *in-vacuo* ELF basin attractors together with the most relevant valence basin populations at Cp **1**, ethylene **4** and the cyanoethylene series **2**. The total population of the V(Cx,Cy) disynaptic basins is given in average number of electrons, e.

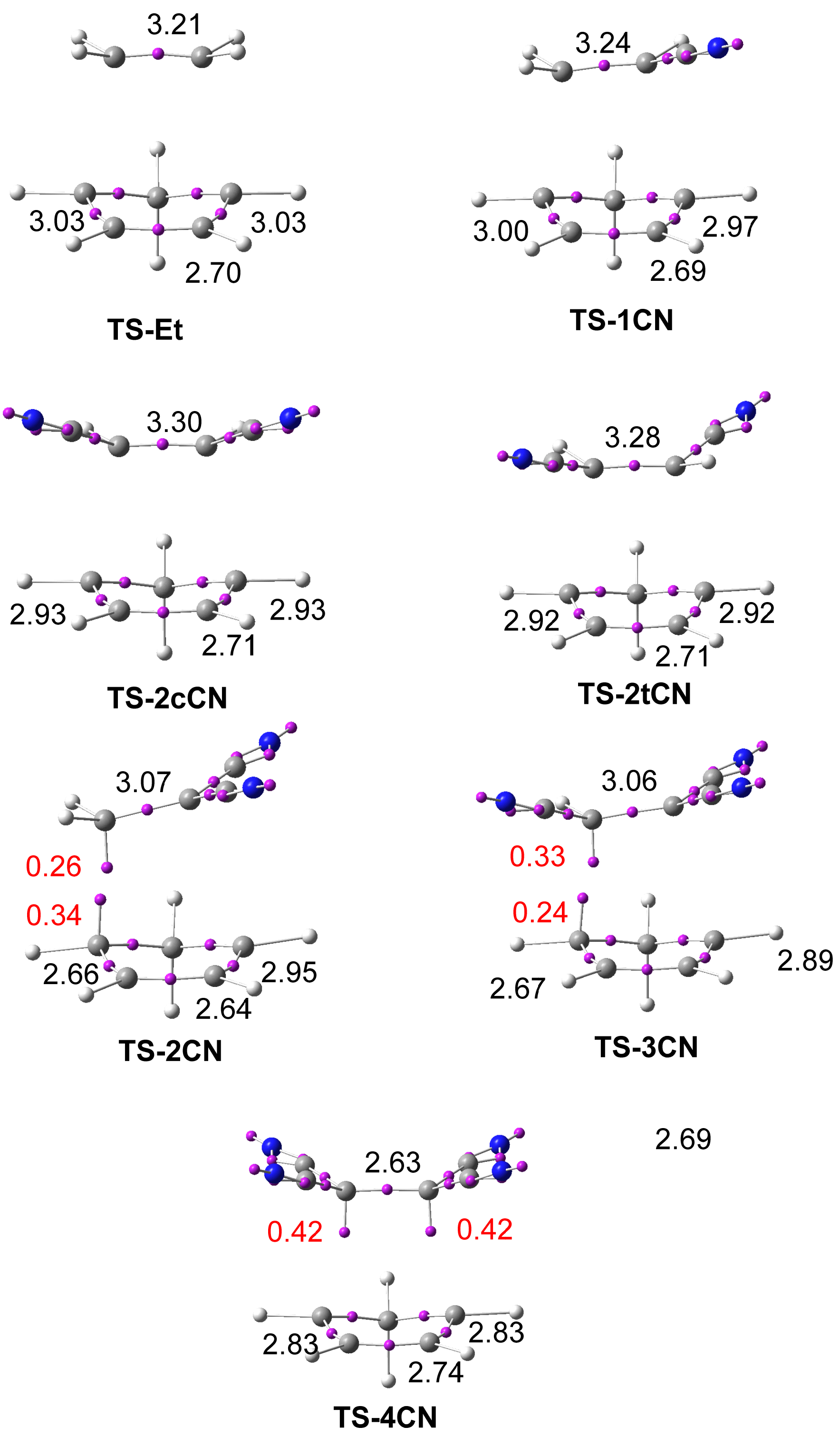

Figure S2. M06-2X/6-311G(d,p) *in-vacuo* the ELF basin attractors together with the most relevant valence basin populations at the TSs involved in the DA reactions of Cp **1** with ethylene **4** and the cyanoethylene series **2**. The population of V(Cx) monosynaptic basins is given in red. The population of the more relevant valence basins is given in average number of electrons, e.

Finally, a QTAIM [2,3] topological analysis of the electron density ρ at the seven TSs was also performed. The contour line maps of the Laplacian $\nabla^2\rho(r)$ of the electron density of three representative TSs are shown in Figure 7, while the most relevant calculated QTAIM parameters of the bonding critical points (CPs) characterizing the two C1−C6 (**CP1**) and C2−C3 (**CP2**) regions at the TSs are given in Table S1. At the seven TS, **CP1** and **CP2** show a positive Laplacian $\nabla^2\rho(r)$ value, indicating the absence of any covalent interaction (see Table 6). At these TSs, the electron density at the two bonding CPs is lower than 0.080. At the asynchronous TSs the electron density at **CP1** is higher than that at the **CP2**, in agreement with the more advanced character of the C1−C6 bond formation than the C2−C3 one (see Table 6). Interesting, except at **TS-2CN**, at the other six TSs the average of the sum of the density of **CP1** and **CP2** is ca. 0.056 e·Å^{-3}.

According the Espinosa criterion [18], the |V(r)|/G(r) values of **CP1** and **CP2**, between 1.19 and 1.82, indicate that they related to non-covalent interactions with somewhat covalent character (see Table S1). Just as the values of electron density in the polar TSs involving non-symmetric ethylenes, the higher |V(r)|/G(r) ratio of **CP1** provides a glimpse into the bond formation asynchronicity as a consequence of the more favorable C1−C6 interaction. Note that the |V(r)|/G(r) value at the **CP1** of the highly asynchronous, 1.8285, which is close to 2, agrees with the presence of the two V(C1) and V(C6) monosynaptic basins topologically characterizing the two C1 and C6 *pseudoradical* centers required for the subsequent C1−C6 single bond formation (see the ELF analysis of **TS-2CN** in Figure S2) [13]. Interestingly, the QTAIM parameters of the non-polar **TS-Et** are very similar to those of the highly polar **TS-4CN**.

Table S1. M06-2X/6-311G(d,p) *in-vacuo* QTAIM parameters of the (3,-1) CPs, as well as the dimensionless |V(r)|/G(r) ratio, at the C1–C6 (**CP1**) and C2–C3 (**CP2**) interacting regions of the of the TSs involved in the DA reactions of Cp **1** with ethylene **4** and the cyanoethylene series **2**. ρ(r) is given in e·Å^{-3}, $\nabla^2\rho$(r) in e·Å^{-5}, and H(r), G(r), and V(r) in hartree·Å^{-3}.

	**Et**		**1CN**		**2cCN**		**2tCN**	
	C1–C6	C2–C3	C1–C6	C2–C3	C1–C6	C2–C3	C1–C6	C2–C3
ρ(r)	0.0552	0.0552	0.0233	0.0454	0.0565	0.0565	0.0566	0.0550
∇^2(r)	0.0436	0.0436	0.1172	0.0469	0.0417	0.0417	0.0413	0.0422
G(r)	0.0224	0.0224	0.0254	0.0192	0.0227	0.0227	0.0226	0.0221
V(r)	-0.0340	-0.0340	-0.0214	-0.0267	-0.0350	-0.0350	-0.0349	-0.0337
H(r)	-0.0115	-0.0115	-0.0039	-0.0075	-0.1230	-0.0123	-0.0123	-0.0116
|V(r)|/G(r)	1.5179	1.5179	0.8425	1.3906	1.5419	1.5419	1.5442	1.5249
	**2CN**		**3CN**		**4CN**			
	C1–C6	C2–C3	C1–C6	C2–C3	C1–C6	C2–C3		
ρ(r)	0.0792	0.0322	0.0713	0.0400	0.0569	0.0569		
∇^2(r)	0.0202	0.0486	0.0284	0.0475	0.0393	0.0393		
G(r)	0.0294	0.0149	0.0269	0.0174	0.0225	0.0225		
V(r)	-0.0537	-0.0177	-0.0467	-0.0229	-0.0351	-0.0351		
H(r)	-0.0244	-0.0028	-0.0198	-0.0055	-0.0127	-0.0127		
|V(r)|/G(r)	1.8265	1.1879	1.7361	1.3161	1.5600	1.5600		

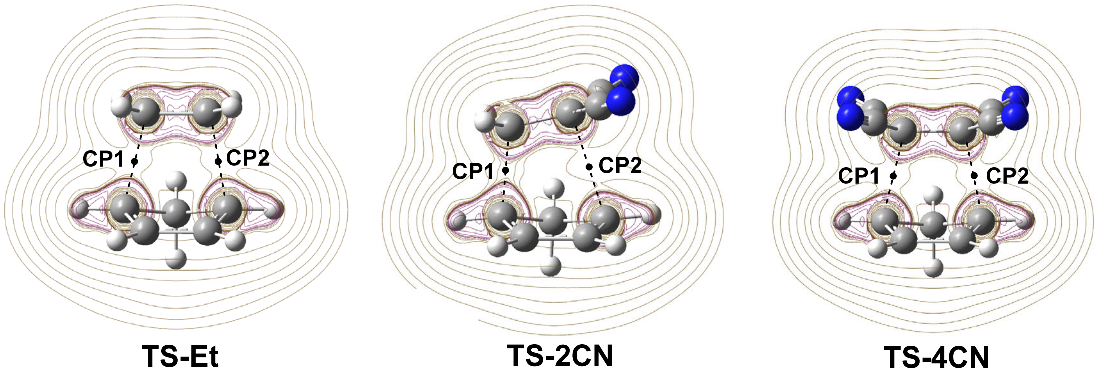

Figure S3. Representations of the contour-line maps of the Laplacian $\nabla^2\rho$(r) of the electron density at the M06-2X/6-311G(d,p) *in-vacuo* optimized **TS-Et**, **TS-2CN** and **TS-4CN**, indicating the position of the (3,−1) CPs **CP1** and **CP2**.

References

1. Blanco, M.A.; Martín Pendás, A.; Francisco, E. Interacting Quantum Atoms: A Correlated Energy Decomposition Scheme Based on the Quantum Theory of Atoms in Molecules. *J. Chem. Theory Comput.* **2005**, *1*, 1096–1109.
2. Bader, R.F.W.: Tang, Y.H.; Tal, Y.; Biegler-König, F.W. Properties of atoms and bonds in hydrocarbon molecules. *J. Am. Chem. Soc*. **1982**, *104*, 946–952.
3. Bader, R.F.W. Atoms in Molecules: A Quantum Theory. Oxford University Press, Oxford, New York, 1994.
4. Wilson, A.L.; Popelier, P.L.A. Exponential Relationships Capturing Atomistic Short-Range Repulsion from the Interacting Quantum Atoms (IQA) Method. *J. Phys. Chem. A*, **2016**, *120*, 9647–9659.
5. Popelier, P.L.A.; Kosov, D.S. Atom–atom partitioning of intramolecular and intermolecular Coulomb energy. *J. Chem. Phys.* **2001**, *114*, 6539–6547.
6. Popelier, P.L.A.; Joubert, L.; Kosov, D.S. Convergence of the Electrostatic Interaction Based on Topological Atoms. *J. Phys. Chem. A* **2001**, *105*, 8254–8261.
7. Martín Pendás, A.; Francisco, E.; Blanco, M.A.; Gatti, C. Bond Paths as Privileged Exchange Channels. *Chem. Eur. J.* **2007**, *13*, 9362–9371.
8. Triestram, L.; Falcioni, F.; Popelier, P.L.A. Interacting Quantum Atoms and Multipolar Electrostatic Study of XH···π Interactions. *ACS Omega* **2023,** *8*, 34844–34851.
9. Becke, A.D.; Edgecombe, K.E. A simple measure of electron localization n atomic and molecular-systems. *J. Chem. Phys.* **1990**, *92*, 5397–5403.
10. Berski, S.; Andrés, J.; Silvi, B.; Domingo, L.R. The joint use of catastrophe theory and electron localization function to characterize molecular mechanisms. A density functional study of the Diels-Alder reaction between ethylene and 1,3-butadiene. *J. Phys. Chem. A* **2003**, *107*, 6014–6024.
11. Domingo, L.R.; Ríos-Gutiérrez, M.; Silvi, B.; Pérez, P. The Mysticism of Pericyclic Reactions: A Contemporary Rationalisation of Organic Reactivity Based on Electron Density Analysis. *Eur. J. Org. Chem.* **2018**, 1107–1120.
12. Domingo, L.R.; Pérez, P.; Sáez, J.A. Origin of the synchronicity in bond formation in polar Diels-Alder reactions: an ELF analysis of the reaction between cyclopentadiene and tetracyanoethylene. *Org. Biomol. Chem.*, **2012**, *10*, 3841-3851.

13. Domingo, L.R. A new C-C bond formation model based on the quantum chemical topology of electron density. *RSC Adv*. **2014**, *4*, 32415-32428.
14. Domingo, L.R.; Sáez, J.A.; Zaragozá, R.J.; Arnó, M. Understanding the Participation of Quadricyclane as Nucleophile in Polar [2σ + 2σ + 2π] Cycloadditions toward Electrophilic π Molecules. *J. Org. Chem.* **2008**, *73*, 8791-8799.
15. Woodward, R.B.; Hoffmann, R. The Conservation of Orbital Symmetry, *Angew. Chem. Int. Ed. Engl.* **1969**, *8*, 781–853.
16. Houk, K.N.; González, J.; Li, Y. Pericyclic reaction transition states: passions and punctilios, 1935-1995. *Acc. Chem. Res.* **1995**, *28*, 81-90.
17. Domingo, L.R.; Ríos-Gutiérrez, M.; Chamorro, E.; Pérez, P. Aromaticity in Pericyclic Transition State Structures? A Critical Rationalisation Based on the Topological Analysis of Electron Density. *ChemistrySelect* **2016**, *1*, 6026–6039.
18. Espinosa, E.; Alkorta, I.; Elguero, J.; Molins, E. From weak to strong interactions: A comprehensive analysis of the topological and energetic properties of the electron density distribution involving X–H···F–Y systems. *J. Chem. Phys*. **2002**, *117*, 5529–5542.

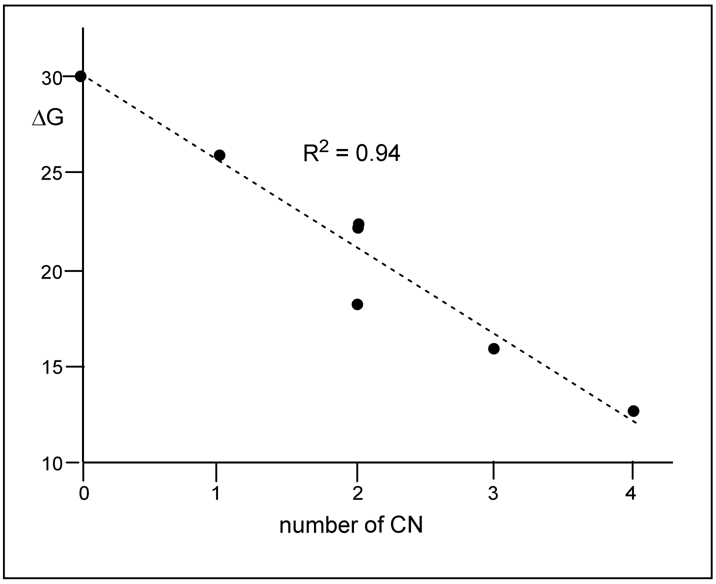

Figure S4. Plot of the computed M06-2X/6-311G(d,p) activation Gibbs free energies, ΔG in kcal mol^{-1}, versus the number of cyano groups on the ethylene, $R^2 = 0.94$, for the DA reactions of Cp **1** with the cyanoethylene series **2**.

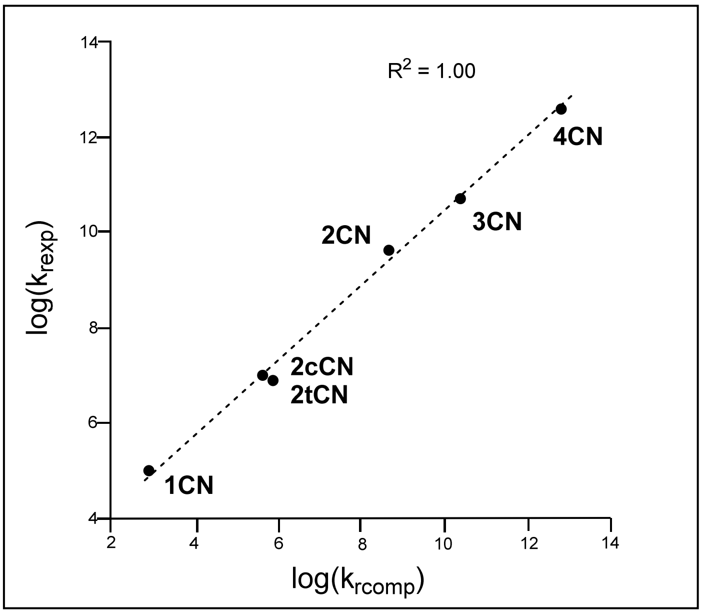

Figure S5. Plot of the logarithm of the experimental relative reaction rate constants log(k_{exp}) versus the logarithm of the computed M06-2X/6-311G(d,p) relative reaction rate constants log(k_{rcomp}), $R^2 = 1.00$, for the DA reactions of Cp **1** with the cyanoethylene series **2**.

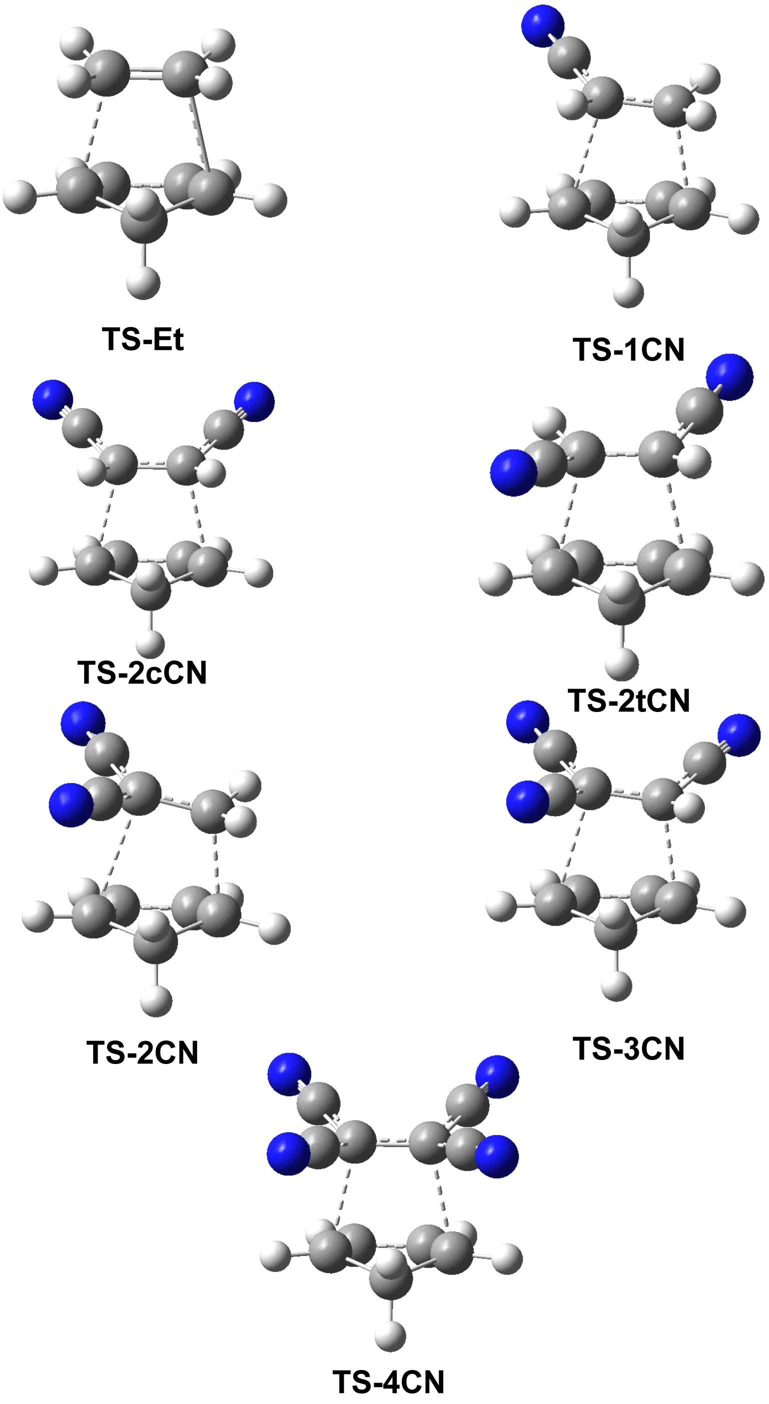

Figure S6. M06-2X/6-311G(d,p) *in-vacuo* optimized geometries of the TSs involved in the DA reactions of Cp **1** with ethylene **4** and the cyanoethylene series **2**.

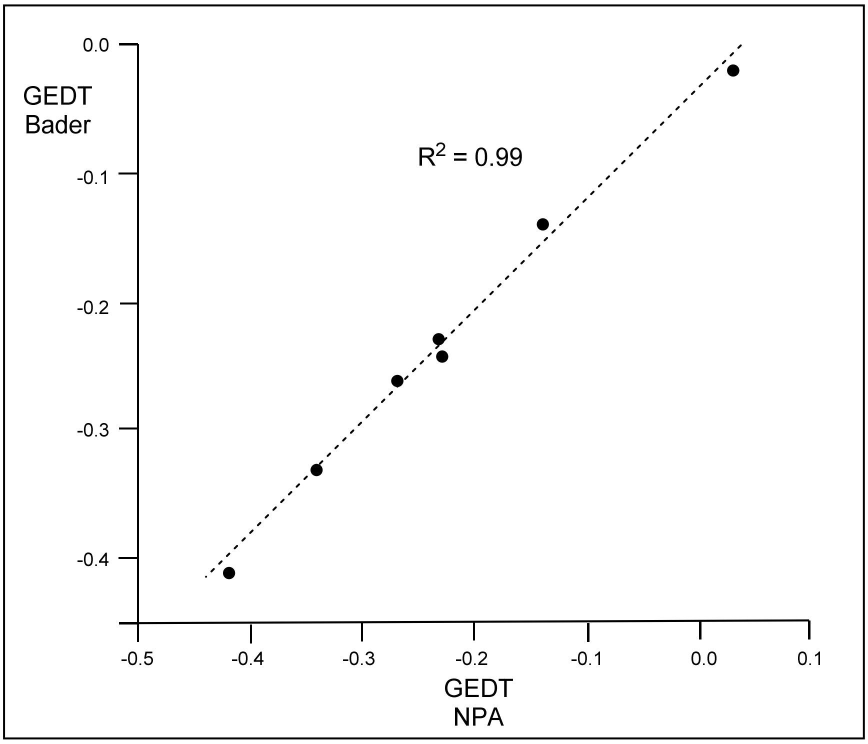

Figure S7. Plot of the *in-vacuo* GEDT obtained by using Bader charges with respect to *in-vacuo* GEDT obtained by using NPA charges. GEDT values, in average number of electrons, e.

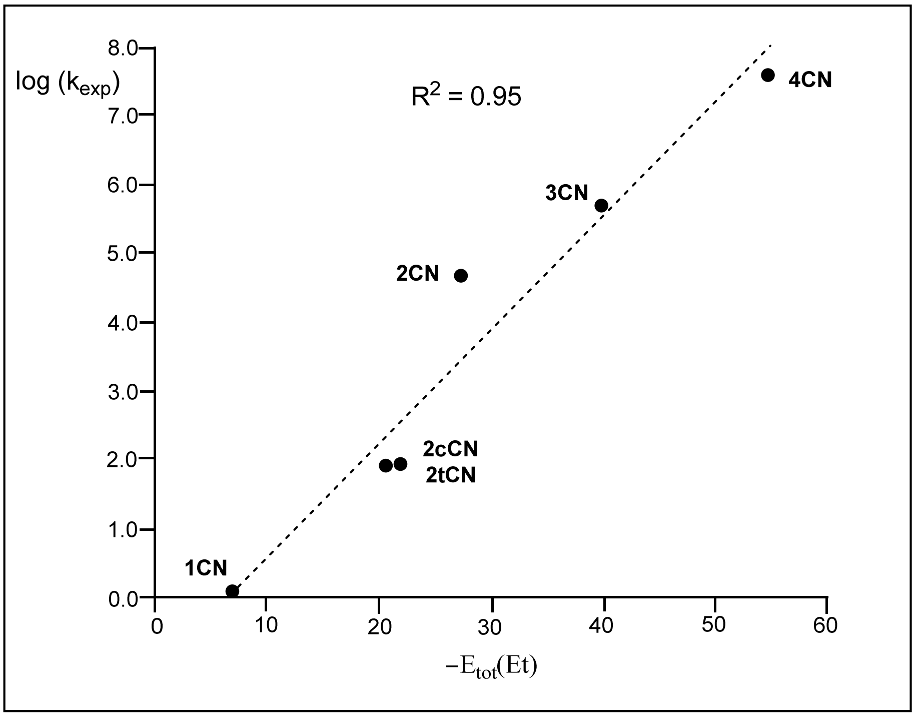

Figure S8. Plot of the logarithm of the experimental reaction rate constants log(k_{exp}) versus the IQA stabilization, $-E_{tot}$(Et) in kcal mol^{-1}, of the ethylene framework, $R^2 = 0.95$, for the DA reactions of Cp **1** with the cyanoethylene series **2**.

Table S2. M06-2X/6-311G(d,p) *in-vacuo* total electronic energies (in Hartree) of reagents, TSs and CAs involved in the DA reactions of Cp **1** with ethylene **4** and the cyanoethylene series **2**.

	Reagents	TSs	CAs
Cp **1**	-194.05702		
Ethylene **4**	-78.562427	-272.594742	-272.669522
**1CN**	-170.800632	-364.839913	-364.908285
**2cCN**	-263.030847	-457.075449	-457.139151
**2tCN**	-263.032198	-457.078071	-457.142207
**2CN**	-263.026264	-457.076905	-457.134582
**3CN**	-355.251855	-549.306069	-549.361562
**4CN**	-447.468236	-641.527854	-641.577167

Table S3. M06-2X/6-311G(d,p) enthalpies (H, in hartree), entropies (S, in cal·mol^{-1}K^{-1}), and Gibbs free energies (G, in hartree), computed at 293.15 K and 1 atm in dioxane, for the stationary points involved in the DA reactions of Cp **1** ethylene **4** and the cyanoethylene series **2**.

	H	S	G
Cp **1**	-193.959901	66.7	-193.991579
Ethylene **4**	-78.507745	52.3	-78.532591
**1CN**	-170.747540	65.0	-170.778442
**2cCN**	-262.978934	75.1	-263.014613
**2tCN**	-262.979814	75.0	-263.015454
**2CN**	-262.973936	74.8	-263.009469
**3CN**	-355.199514	84.7	-355.239776
**4CN**	-447.415019	95.0	-447.460151
**TS-Et**	-272.440348	76.4	-272.476646
**TS-1CN**	-364.688046	85.6	-364.728710
**TS-2cCN**	-456.925660	94.8	-456.970723
**TS-2tCN**	-456.927151	94.4	-456.972018
**TS-2CN**	-456.926714	95.3	-456.971992
**TS-3CN**	-549.156682	103.9	-549.206039
**TS-4CN**	-641.378297	112.1	-641.431563
**CA-Et**	-272.509958	72.9	-272.544579
**CA-1CN**	-364.751216	82.0	-364.790184
**CA-2cCN**	-456.984857	91.0	-457.028114
**CA-2tCN**	-456.986709	91.3	-457.030076
**CA-2CN**	-456.978688	90.8	-457.021820
**CA-3CN**	-549.207624	99.8	-549.255053
**CA-4CN**	-641.423972	108.4	-641.475474

Table S4. Distances between the C1–C6 and C2–C3 interacting carbons at the M06-2X/6-311G(d,p) optimized TSs in dioxane, geometrical asynchronicity Δl, and average of the two C–C interacting distances lm, in angstroms, Å, and GEDT values, in average number of electrons, e.

	C1–C6	C2–C3	Δl	lm	GEDT[a]	GEDT[b]
Ethylene **4**	2.220	2.220	0.00	2.22	0.03	-0.02
**1CN**	2.108	2.355	0.25	2.23	-0.16	-0.14
**2cCN**	2.226	2.226	0.00	2.23	-0.25	-0.23
**2tCN**	2.226	2.238	0.01	2.23	-0.26	-0.24
**2CN**	2.009	2.593	0.58	2.30	-0.30	-0.26
**3CN**	2.090	2.465	0.38	2.28	-0.37	-0.33
**4CN**	2.247	2.247	0.00	2.25	-0.46	-0.41

[a] GEDT values with natural charges in dioxane.

[b] GEDT values with Bader charges in *in-vacuo*.

Table S5. Total intra- ($\sum E_{intra}^{A}$) and interatomic ($\sum V_{inter}^{A}$) IQA energies, in hartree, at the diene and ethylene frameworks of the TSs and at the reagents involved in the DA reactions of Cp **1** with ethylene **4** and the cyanoethylene series **2**.

	TS		reagents	
	$\sum E_{intra}^{A}$	$\sum V_{inter}^{A}$	$\sum E_{intra}^{A}$	$\sum V_{inter}^{A}$
**Cp**	-190.699391	-3.346147	-190.754475	-3.302718
**Et**	-77.040088	-1.509279	-77.092859	-1.469548
**Cp**	-190.694280	-3.334363		
**1CN**	-167.830729	-2.980904	-167.885475	-2.915097
**Cp**	-190.687136	-3.324303		
**2cCN**	-258.633670	-4.430046	-258.712430	-4.318321
**Cp**	-190.687837	-3.323830		
**2tCN**	-258.630012	-4.436618	-258.704100	-4.327995
**Cp**	-190.688841	-3.318591		
**2CN**	-258.639592	-4.429818	-258.686337	-4.339788
**Cp**	-190.681018	-3.309782		
**3CN**	-349.447705	-5.867486	-349.535004	-5.716685
**Cp**	-190.671793	-3.300780		
**4CN**	-440.268125	-7.287077	-440.383764	-7.084255

M06-2X/6-311G(d,p) *in-vacuo* computed total energies, single imaginary frequencies of TSs, and Cartesian coordinates of the stationary points involved in the DA reactions of Cp **1** with ethylene **4** and the cyanoethylene series **2**.

Cp **1**

E(RM062X) = -194.057020221 A.U.

6	-1.177376	-0.274812	-0.000091
6	-0.730456	0.990361	0.000311
6	0.739634	0.983509	-0.000423
6	1.174735	-0.285649	0.000358
1	-2.209420	-0.596041	-0.000756
1	-1.340555	1.883631	0.000526
1	1.358029	1.871053	-0.000517
1	2.203791	-0.616296	-0.000008
6	-0.005624	-1.213838	-0.000082
1	-0.008760	-1.869530	0.878750
1	-0.008563	-1.870240	-0.878434

Ethylene **4**

E(RM062X) = -78.5624266445 A.U.

6	0.000000	-0.000000	0.662137
6	0.000000	-0.000000	-0.662137
1	-0.000000	0.923343	-1.230302
1	0.000000	0.923343	1.230302
1	-0.000000	-0.923343	1.230302
1	0.000000	-0.923343	-1.230302

1CN

E(RM062X) = -170.800632180 A.U.

6	0.591022	0.510404	-0.000000
6	1.592851	-0.363285	-0.000000
6	-0.782230	0.095173	0.000000
7	-1.886341	-0.225750	0.000000
1	2.620224	-0.022793	0.000000
1	1.410917	-1.430749	0.000000
1	0.763383	1.580040	0.000000

2cCN

E(RM062X) = -263.030847119 A.U.

6	0.810727	0.327862	-0.676153
6	0.953299	0.398113	0.649821
6	1.360240	-0.710853	1.455481
7	1.685613	-1.586273	2.125399
6	1.049902	-0.863733	-1.429387

7	1.235556	-1.808013	-2.057787
1	0.758459	1.327209	1.170400
1	0.499598	1.199625	-1.237774

2tCN

E(RM062X) = -263.032198126 A.U.

6	0.810727	0.327862	-0.676153
6	0.953299	0.398113	0.649821
6	1.360240	-0.710853	1.455481
7	1.685613	-1.586273	2.125399
6	1.049902	-0.863733	-1.429387
7	1.235556	-1.808013	-2.057787
1	0.758459	1.327209	1.170400
1	0.499598	1.199625	-1.237774

2CN

E(RM062X) = -263.026263503 A.U.

6	-0.000027	1.736820	0.000005
6	0.000001	0.401590	-0.000048
6	1.226343	-0.351147	-0.000007
7	2.211160	-0.941977	0.000017
6	-1.226309	-0.351204	-0.000010
7	-2.211155	-0.941983	0.000018
1	-0.932660	2.285660	0.000062
1	0.932581	2.285705	0.000052

3CN

E(RM062X) = -355.251855126 A.U.

6	0.825896	0.402680	-0.648888
6	0.962831	0.478980	0.685146
6	1.462827	-0.602071	1.468071
7	1.863208	-1.458995	2.121250
6	1.171746	-0.774161	-1.393078
7	1.447504	-1.714375	-1.991990
6	0.316555	1.521902	-1.389937
7	-0.091639	2.421826	-1.975139
1	0.688927	1.387831	1.207127

4CN

E(RM062X) = -447.468235761 A.U.

6	0.756121	0.365728	-0.664012
6	0.998642	0.464471	0.663926
6	1.347247	-0.684122	1.443807
7	1.627482	-1.601568	2.075621

6	0.916795	1.721604	1.343496
7	0.853176	2.726873	1.895596
6	0.838596	-0.891257	-1.343766
7	0.902856	-1.896984	-1.894957
6	0.408102	1.514404	-1.444014
7	0.128302	2.432072	-2.075698

TS-Et

E(RM062X) = -272.594741720 A.U.
Freq = -494.6610 cm^{-1}

6	-0.578816	0.053731	1.152881
6	-0.578816	-1.262204	0.701277
6	-0.578816	-1.262204	-0.701277
6	-0.578816	0.053731	-1.152881
6	1.527336	0.595737	-0.690092
6	1.527336	0.595737	0.690092
1	-0.691746	0.346076	2.189024
1	-0.461625	-2.136966	1.327045
1	1.444431	1.529343	1.234055
1	1.444431	1.529343	-1.234055
1	-0.461625	-2.136966	-1.327045
1	-0.691746	0.346076	-2.189024
6	-1.029431	0.909878	-0.000000
1	-2.129164	0.917384	-0.000000
1	-0.672462	1.937700	-0.000000
1	1.979829	-0.219210	-1.238314
1	1.979829	-0.219210	1.238314

TS-1CN

E(RM062X) = -364.839913295 A.U.
Freq = -478.0355 cm^{-1}

6	-1.613269	0.428291	0.604757
6	-1.124885	-0.766226	1.137643
6	-0.474314	-1.487794	0.126910
6	-0.569035	-0.778628	-1.060851
6	0.930151	0.921514	-0.471537
6	0.173548	1.561505	0.502051
1	-2.284001	1.093788	1.133759
1	-1.130036	-1.028087	2.187226
1	0.121659	-2.377058	0.277172
1	-0.202417	-1.120862	-2.019645
6	-1.648559	0.249032	-0.892526
1	-2.607130	-0.224757	-1.146831
1	-1.543771	1.154491	-1.487423
6	2.031993	0.095110	-0.090476
7	2.906826	-0.581335	0.233777
1	-0.351054	2.470831	0.236528
1	0.470126	1.483305	1.538773
1	0.945057	1.280877	-1.491820

TS-2cCN

E(RM062X) = -457.075449225 A.U.
Freq = -456.6286 cm^{-1}

6	-1.497538	-0.423351	1.151790
6	-1.380268	-1.736381	0.699616
6	-1.380268	-1.736381	-0.699616
6	-1.497538	-0.423351	-1.151790
6	0.569421	0.247992	-0.698743
6	0.569421	0.247992	0.698743
1	-1.614592	-0.142483	2.190643
1	-1.177468	-2.593900	1.326070
1	-1.177468	-2.593900	-1.326070
1	-1.614592	-0.142483	-2.190643
6	-1.998814	0.402132	-0.000000
1	-3.095915	0.344972	-0.000000
1	-1.712633	1.452617	-0.000000
6	1.308832	-0.728573	1.441255
7	1.883376	-1.515127	2.053775
6	1.308832	-0.728573	-1.441255
7	1.883376	-1.515127	-2.053775
1	0.414931	1.186721	1.214086
1	0.414931	1.186721	-1.214086

TS-2tCN

E(RM062X) = -457.078070984 A.U.
Freq = -445.3566 cm^{-1}

6	-1.505015	-0.404827	1.149002
6	-1.361939	-1.720747	0.707781
6	-1.357660	-1.731136	-0.690992
6	-1.500855	-0.424296	-1.154446
6	0.560131	0.302246	-0.697108
6	0.556223	0.283578	0.700911
1	-1.633287	-0.119223	2.185221
1	-1.144956	-2.569128	1.341987
1	-1.156724	-2.595094	-1.309825
1	-1.619533	-0.141673	-2.192647
6	-2.018959	0.399731	-0.009528
1	-3.114316	0.312313	-0.013291
1	-1.763661	1.457677	-0.026071
6	1.301375	-0.732079	1.382628
7	1.886249	-1.562965	1.923295
6	0.410470	1.551865	-1.379237
7	0.257415	2.555094	-1.923242
1	1.092508	-0.464237	-1.242363
1	0.426717	1.210351	1.245189

TS-2CN

E(RM062X) = -457.076905062 A.U.

Freq = -435.5170 cm^{-1}

6	1.691644	-0.629924	0.782699
6	2.085452	0.599924	0.239282
6	1.475796	0.771440	-1.015079
6	0.752641	-0.364496	-1.313458
6	-0.971254	0.129262	0.478646
6	-0.115673	-0.142593	1.555227
1	2.182894	-1.078366	1.637755
1	2.661367	1.353146	0.760854
1	1.478646	1.685263	-1.591986
1	0.154502	-0.524537	-2.200880
6	1.146790	-1.447975	-0.364880
1	1.992735	-1.990796	-0.809845
1	0.369522	-2.173107	-0.130112
6	-1.204358	1.484050	0.091835
7	-1.339765	2.591992	-0.193168
6	-1.838329	-0.881317	-0.036550
7	-2.503304	-1.732009	-0.439355
1	-0.250534	-1.086949	2.069052
1	0.176083	0.685244	2.186495

TS-3CN

E(RM062X) = -549.306069309 A.U.
Freq = -415.6052 cm^{-1}

6	0	-1.426484	-0.388074	1.116808
6	0	-1.313034	-1.709249	0.661331
6	0	-1.402683	-1.722363	-0.735898
6	0	-1.616456	-0.426638	-1.180646
6	0	0.630511	0.309223	-0.681027
6	0	0.517440	0.278984	0.723613
1	0	-1.542519	-0.126140	2.161363
1	0	-1.052825	-2.556922	1.280822
1	0	-1.211960	-2.578512	-1.367467
1	0	-1.732026	-0.123174	-2.213200
6	0	-2.023851	0.411733	-0.011411
1	0	-3.116756	0.343835	0.080847
1	0	-1.759187	1.466308	-0.066422
6	0	1.289168	-0.665068	1.484489
7	0	1.880730	-1.422452	2.115088
6	0	1.324568	-0.727847	-1.380977
7	0	1.864626	-1.583796	-1.928171
6	0	0.441574	1.552601	-1.363015
7	0	0.249434	2.561634	-1.883160
1	0	0.371921	1.231618	1.219901

TS-4CN

E(RM062X) = -641.527853908 A.U.
Freq = -386.2558 cm^{-1}

6	0	-1.510533	-0.411141	1.149413

6	0	-1.353498	-1.726952	0.697023
6	0	-1.353498	-1.726952	-0.697023
6	0	-1.510533	-0.411141	-1.149413
6	0	0.555633	0.289396	-0.712634
6	0	0.555633	0.289396	0.712634
1	0	-1.617038	-0.123410	2.187946
1	0	-1.133112	-2.578838	1.325402
1	0	-1.133112	-2.578838	-1.325402
1	0	-1.617038	-0.123410	-2.187946
6	0	-2.024237	0.403843	0.000000
1	0	-3.119625	0.318375	0.000000
1	0	-1.775142	1.464045	0.000000
6	0	1.296995	-0.726395	1.408374
7	0	1.864720	-1.556703	1.964465
6	0	0.413550	1.538211	1.408923
7	0	0.257761	2.531101	1.967133
6	0	1.296995	-0.726395	-1.408374
7	0	1.864720	-1.556703	-1.964465
6	0	0.413550	1.538211	-1.408923
7	0	0.257761	2.531101	-1.967133

CA-Et

E(RM062X) = -272.669521629 A.U.

6	0.223081	0.251602	1.124514
6	-1.125093	0.780175	0.666972
6	-1.125093	0.780175	-0.666972
6	0.223081	0.251602	-1.124514
6	0.223081	-1.270421	-0.777350
6	0.223081	-1.270421	0.777350
1	0.509109	0.480485	2.149347
1	-1.960691	0.993175	1.321350
1	1.122812	-1.747613	1.171556
1	1.122812	-1.747613	-1.171556
1	-1.960691	0.993175	-1.321350
1	0.509109	0.480485	-2.149347
6	1.127585	0.791418	-0.000000
1	1.197978	1.879562	0.000000
1	2.125766	0.345052	-0.000000
1	-0.642265	-1.780746	-1.200645
1	-0.642265	-1.780746	1.200645

CA-1CN

E(RM062X) = -364.908285113 A.U.

6	-1.482001	-0.434806	-0.625868
6	-1.129910	1.004077	-0.956609
6	-0.405176	1.487158	0.053065
6	-0.265472	0.379779	1.081768
6	0.688064	-0.694772	0.444392
6	-0.152235	-1.238891	-0.755993
1	-2.316106	-0.868589	-1.173106

1	-1.334328	1.487990	-1.902587
1	0.107390	2.438217	0.099530
1	0.028905	0.676308	2.085849
6	-1.610924	-0.346144	0.906731
1	-2.454767	0.265029	1.225423
1	-1.643902	-1.323341	1.395967
6	1.982397	-0.142723	0.054990
7	2.996704	0.301634	-0.252064
1	-0.327556	-2.309289	-0.639525
1	0.340431	-1.071106	-1.712501
1	0.874553	-1.488728	1.170544

CA-2cCN

E(RM062X) = -457.139151054 A.U.

6	-1.236734	-0.298571	1.113876
6	-1.416197	-1.726406	0.633194
6	-1.391844	-1.708213	-0.699456
6	-1.195602	-0.267823	-1.134085
6	0.285838	0.102262	-0.764435
6	0.257035	0.081135	0.808837
1	-1.525104	-0.083534	2.139921
1	-1.428801	-2.594374	1.277697
1	-1.380541	-2.558314	-1.367375
1	-1.446384	-0.024861	-2.163774
6	-1.938194	0.478409	-0.012926
1	-3.011376	0.295757	-0.035133
1	-1.737260	1.552847	0.005466
6	1.206396	-0.828610	1.440761
7	1.931225	-1.546264	1.968120
6	1.257760	-0.791251	-1.385187
7	2.002164	-1.494336	-1.904850
1	0.465671	1.085547	1.182451
1	0.507976	1.116119	-1.103104

CA-2tCN

E(RM062X) = -457.142207271 A.U.

6	-1.225252	-0.260365	1.137811
6	-1.304611	-1.723545	0.740270
6	-1.338800	-1.775821	-0.591265
6	-1.281717	-0.351284	-1.108713
6	0.188645	0.130384	-0.826620
6	0.214109	0.225622	0.743229
1	-1.487461	-0.013775	2.163681
1	-1.226448	-2.553339	1.429132
1	-1.299345	-2.658735	-1.214814
1	-1.595672	-0.182126	-2.135661
6	-2.036354	0.394263	0.006110
1	-3.090639	0.124483	0.043961
1	-1.923921	1.480162	-0.044551
6	1.276250	-0.574879	1.343796

7	2.098727	-1.221002	1.818456
6	0.450443	1.429319	-1.442024
7	0.620415	2.459799	-1.920601
1	0.922558	-0.584561	-1.197872
1	0.353255	1.262851	1.052937

CA-2CN

E(RM062X) = -457.134581523 A.U.

6	1.568047	-0.710829	0.762459
6	2.159526	0.563451	0.188312
6	1.461650	0.881161	-0.902305
6	0.396740	-0.183340	-1.082990
6	-0.649967	0.056837	0.090420
6	0.192259	-0.309190	1.371097
1	2.196434	-1.272988	1.449296
1	2.932678	1.153640	0.662064
1	1.535395	1.780888	-1.497010
1	-0.083697	-0.253567	-2.055664
6	1.117617	-1.422013	-0.527169
1	1.953725	-1.718897	-1.158651
1	0.453973	-2.272049	-0.352275
6	-1.137574	1.438726	0.107664
7	-1.487548	2.531984	0.117969
6	-1.787148	-0.856665	-0.069765
7	-2.641091	-1.612011	-0.206175
1	-0.274134	-1.144467	1.893258
1	0.259201	0.538809	2.050082

CA-3CN

E(RM062X) = -549.361562170 A.U.

6	-1.114020	-1.194407	-0.770403
6	-1.820325	-1.025436	0.561889
6	-0.889817	-1.035009	1.516070
6	0.454800	-1.215183	0.839604
6	0.745394	0.161505	0.098401
6	-0.361135	0.154651	-1.042363
1	-1.722228	-1.505242	-1.616131
1	-2.874670	-0.815287	0.676167
1	-1.022166	-0.832118	2.569465
1	1.293130	-1.524751	1.458426
6	0.071030	-2.086560	-0.366076
1	-0.247403	-3.081317	-0.059744
1	0.853714	-2.157098	-1.124987
6	-1.246612	1.312663	-1.033597
7	-1.974192	2.200553	-1.039857
6	0.658354	1.294306	1.022214
7	0.585317	2.155758	1.776476
6	2.082749	0.129336	-0.505194
7	3.117179	0.046919	-0.995886
1	0.138981	0.118001	-2.011601

CA-4CN

E(RM062X) = -641.577166570 A.U.

6	-0.513489	1.123985	-1.228853
6	-1.949952	0.666691	-1.076566
6	-1.949975	-0.665892	-1.076917
6	-0.513538	-1.123142	-1.229551
6	0.193314	-0.805495	0.158952
6	0.193373	0.805468	0.159448
1	-0.341612	2.154236	-1.529622
1	-2.785187	1.326339	-0.887796
1	-2.785231	-1.325620	-0.888514
1	-0.341709	-2.153217	-1.530951
6	0.077020	0.000679	-2.093393
1	-0.341061	0.001006	-3.098028
1	1.168537	0.000675	-2.138686
6	-0.549596	1.380573	1.282481
7	-1.159114	1.853074	2.131765
6	1.551045	1.358812	0.154311
7	2.601419	1.816650	0.095259
6	-0.549718	-1.381181	1.281646
7	-1.159278	-1.854538	2.130425
6	1.550951	-1.358919	0.153477
7	2.601210	-1.817027	0.094464
